# *JORT* celebrates 18 years of publication

**DOI:** 10.1186/s10195-018-0517-8

**Published:** 2019-01-03

**Authors:** Marco d’Imporzano, Luca Pierannunzii

**Affiliations:** 1 Istituto Auxologico Italiano, Clinica “Capitanio”, 20122 Milan, Italy; 2 Gaetano Pini Orthopaedic Institute, ASST G. Pini-CTO, Piazza Cardinal A. Ferrari, 1, 20122 Milan, Italy

The *Journal of Orthopaedics and Traumatology* (*JORT*), founded in 2000 by Francesco Pipino, has been publishing regularly for 18 years. The initial vision was an English-language journal that could communicate the results of Italian musculoskeletal research to the rest of the world whilst also reciprocally absorbing the newest orthopaedic developments circulating internationally. This objective was immediately and enthusiastically embraced by the Italian Society of Orthopaedics and Traumatology (SIOT) and the journal’s distinguished international editorial board, whose mission was to enhance a bidirectional exchange of findings, techniques and knowledge between Italy and the rest of the world and, more recently, promote international cooperation in musculoskeletal research.

The collaboration with a scientific publisher like Springer allowed us to exploit a submissions management platform that helped editors shepherd manuscripts through a rigorous double-blind peer-review system, which guarantees the highest scientific quality. Making *JORT*’s contents open to all potential readers (open-access policy) was a choice that SIOT has made since the very beginning of its editorial experience, permitting authors to retain the right to store, reuse and distribute their papers.

In 2017, the journal entered a new phase of its life; the open-access publication, granted for free by SIOT to all authors before then, was replaced by an “author-pays” model, whereby article processing charges (APCs) are waived only for those authors whose full membership is confirmed by the society or for those scientific referees whose curriculum and commitment are considered outstanding. The publisher offers a free open-access support funding service [1] to help authors discover and apply for article processing charge funding. Although we saw an initial reduction in submission numbers before these changes were absorbed by authors, nowadays we are back on a positive trajectory and are returning to consistent and regular publications. All accepted papers are copyedited and produced consecutively under copyright license CC-BY 4.0; issues and volumes were buried with the printed editions; no more online first, or—if you prefer—online first for all and forever.

Two-thirds of 2018 production come from international institutions, clearly displaying *JORT*’s diversity in authorship. Often, the author team is made up of academics from different countries who collaborate with a profitable synergy towards the same objective. One-quarter of our associate editors is based in countries other than Italy, as well as half of the advisors and many reviewers. Very soon, a renewal is planned in the editorial structure of the journal, to satisfy the ambitions of those who have contributed more (e.g. peer-reviewing) to *JORT*’s success and to allow some rest to those who have been proud defenders of *JORT* from the very early days of this enthusiastic editorial experience. This renewal will represent a further opportunity to increase the international diversity of the journal. *JORT*’s submissions previously outnumbered the referees and associate editors dedicated to each research field; at the same time, musculoskeletal research has developed in so many directions that regular updates and new designations were required. The recent history of our journal has taught us that self-nominations with strict requirements in terms of both proficiency and commitment allowed us to assign *JORT*’s key positions more wisely than third-party nominations did in the past. These features should be confirmed by high author-level scientometrics and hopefully might associate with countries of origin marked by high scientific output (USA, UK, India, China, Japan etc.), although such information cannot be considered a criterion of preference.

Back in 2016, three thematic series were announced to *JORT* readers:

- *Commentaries*, brief observations written by editors or advisors that endorse the contents of a cutting-edge research paper published at the same time.
- *Emerging topics*, narrative reviews or mini-reviews commissioned by editors from top scientists in a novel field of research (level of evidence V).
- *Controversies in orthopaedics and traumatology*, narrative reviews or mini-reviews commissioned by editors from top orthopaedic surgeons to illustrate their preferred surgical technique (or treatment protocol, aetiopathogenetic model etc.). Obviously, “controversies” are published in a minimum number of two per subject, as a topic is defined as “controversial” only if it may be interpreted or managed from two perspectives or more.

*JORT* has exerted a remarkable teaching effect thanks to these commissioned contributions, and it will be our commitment to add new thematic contents as of January 2019. The prevailing focus of next year’s thematic contributions will be orthobiologics and regenerative medicine, as this field is novel enough to catch readers’ attention and has gathered enough experience to benefit from a critical review.

At the time of writing, *JORT*’s articles have been downloaded 226,435 times and received 87 Altmetric mentions [2].

According to Google Scholar, the h5-index of *JORT* is 23, which means that at least 23 articles published from 2013 to 2017 obtained a minimum of 23 citations in the database [3]. According to Scopus database, our journal displays a Scimago Journal Rank (SJR) of 1175 and an h-index of 28 [4], and ranks in the first quartile (Q1) in orthopedics and sports medicine, medicine and surgery.

As for citations, in 2017 the Scopus CiteScore reached 2.06. Presently the attribution of a journal impact factor would represent a prestigious acknowledgement for all the efforts from SIOT to guarantee *JORT*’s 18-year publication, for all the scientific and personal efforts that editors have put into managing a growing number of submissions and into aiming at continuous quality improvement, as well as for all the academic efforts that authors have contributed to provide sound reports of worldwide orthotraumatological research.

In conclusion, we thank you all for your continuous commitment.
